# Life-course socioeconomic circumstances and changes in leisure-time physical activity among young and early midlife employees

**DOI:** 10.1186/s12939-026-02769-3

**Published:** 2026-01-30

**Authors:** Ville Päivärinne, Jatta Salmela, Jouni Lahti, Olli Pietiläinen, Anni Karjala, Anne Kouvonen, Ossi Rahkonen, Tea Lallukka

**Affiliations:** 1 Department of Public Health, University of Helsinki, P.O. Box 20, Helsinki, FI-00014 Finland; 2 Finnish Institute for Health and Welfare, Helsinki, Finland; 3 Faculty of Social Sciences, University of Helsinki, Helsinki, Finland; 4 Centre for Public Health, Queen’s University Belfast, Belfast, Northern Ireland

## Abstract

**Background:**

While socioeconomic differences in leisure-time physical activity (LTPA) are well established, less is known about the contribution of socioeconomic factors to changes in LTPA. This study examined the associations between life-course socioeconomic position (SEP) and changes in LTPA over a 5-year follow-up among young and early midlife employees.

**Methods:**

This study used the Helsinki Health Study follow-up survey data, comprised of employees of the City of Helsinki aged 19–39 in 2017 (Phase 1). The follow-up was completed in 2022 (Phase 2). The analytical sample comprised 2,615 participants (80% women, which correspond to the target population). LTPA was measured as metabolic equivalent (METs, continuous and dichotomized) across three intensity levels (light, moderate, vigorous). SEP indicators included parental and own education, occupational class, childhood and current economic difficulties, household income, and household wealth. Marginal effects were calculated from generalized linear mixed models to examine associations between SEP and LTPA changes, adjusting for sociodemographic and health-related confounders.

**Results:**

During the follow-up, total (-2.84 MET [95% CI: -4.32 to -1.35]) and vigorous (-6.68 MET [95% CI: -8.22 to -5.43]) LTPA decreased, while light LTPA increased (1.43 MET [95% CI: 0.69 to 2.17]). Engagement in vigorous LTPA declined across all SEP groups, indicating similar disengagement patterns and persistent disparities between high and low SEP groups over time. The largest decline in vigorous LTPA activity levels was observed among participants with high household income (-11.42 MET [95% CI: -16.15 to -6.69]) and higher own education (-9.15 MET [95% CI: -13.60 to -4.69]). While this led to narrowing differences in these and lower SEP groups over the follow-up, disparities in vigorous LTPA between high and low SEP groups remained. Moderate LTPA increased among the higher-educated, while light LTPA did so in higher-income groups, partially offsetting the decline in vigorous LTPA within these groups.

**Conclusion:**

Our findings raise concerns about the long-term consequences of overall declining vigorous LTPA, particularly among lower SEP employees with lower initial engagement and no compensatory increases. Persistent SEP differences suggest a multi-dimensional challenge, emphasizing the need for targeted interventions to promote LTPA of all intensities and reduce health disparities.

**Clinical trial number:**

Not applicable.

**Supplementary Information:**

The online version contains supplementary material available at 10.1186/s12939-026-02769-3.

## Introduction

The health benefits of leisure-time physical activity (LTPA) are well known: regular engagement can add 1–2 disease-free years, with the greatest benefits observed among individuals at higher risk of health problems, particularly those with lower socioeconomic status [1]. This emphasizes the potential of policy interventions promoting physical activity to enhance life expectancy and public health [2]. Physical activity can be categorized by its intensity—measured in terms of energy expenditure—into light, moderate and vigorous levels. To promote health, the World Health Organization (WHO) recommends that all adults engage in at least 150 min of moderate intensity or 75 min of vigorous-intensity physical activity per week and reduce sedentary behavior [3]. A recent study suggests that even short bouts of vigorous or moderate LTPA each day may help mitigate the cardiovascular risks of prolonged sedentary behavior [4].

Early-life socioeconomic circumstances shape long-term health and behavior, making life-course indicators essential in understanding socioeconomic differences in LTPA [5, 6]. A systematic review showed that a less advantaged childhood SEP is associated with lower LTPA in adulthood [7], while a recent cohort study demonstrated an association between educational attainment and LTPA levels from childhood to midlife [6]. Socioeconomic disadvantages, such as low income, education, or occupational class, have consistently been associated with lower engagement in physical activity [3, 8]. Those with lower SEP are also less likely to meet physical activity guidelines and report greater barriers to an active lifestyle compared to those from higher SEP groups [9, 10]. Higher SEP is associated with greater LTPA participation, particularly participation in moderate-to-vigorous intensity exercise [9]. Cross-sectional studies have consistently shown that low education is associated with lower LTPA levels, whereas higher education and income are positively associated with LTPA among young and midlife adults [11, 12]. Longitudinal studies have further confirmed persistent socioeconomic differences, with education, occupational class, and income shaping physical activity patterns over time [11, 13]. Notably, participation in moderate and vigorous level of LTPA is more common in high-income households [14].

While strong evidence supports the association between SEP and LTPA, some aspects of this association remain unclear. An umbrella literature review by O’Donoghue et al. (2018) found inconsistent evidence regarding the association between specific life-course SEP components (e.g., education and income) and overall physical activity or LTPA [15]. This may be partly due to methodological differences, such as the tendency of previous studies to examine individual SEP factors within specific age groups rather than employing a comprehensive life-course approach [16]. In addition, few studies have simultaneously examined the association between three key SEP indicators—education, income, and occupational class—and LTPA within the same data [17]. Among these, some have found a positive association between higher SEP and LTPA [18, 19], while others have reported a negative association [20, 21], highlighting the complexity of these relationships.

Regular LTPA can extend disease free years, with the greatest benefits seen among individuals at higher health risk, particularly those with lower SEP [1]. This highlights the role of physical activity in mitigating health disparities and addressing life-course socioeconomic differences in health outcomes. In sum, while there is evidence of the socioeconomic differences in LTPA, the changes in these differences—with different intensities and simultaneously assessing several SEP indicators —have been less studied [13, 22, 23].

The aim of this study was to examine how childhood and adulthood SEP indicators are associated with different intensities of LTPA (light, moderate and vigorous) and their changes over a 5-year follow-up among young and early midlife Finnish employees.

### Design and participants

This study utilized data from the Helsinki Health Study, targeting the City of Helsinki employees aged 19–39 years (Phase 1 in 2017, *n* = 5,898). More detailed description of the data and the participants can be found elsewhere [24]. Phase 2 survey was collected in 2022 (*n* = 3,520, response rate 60%). Participants who completed the survey via telephone interview (*n* = 285) were excluded, as LTPA data was not collected through this method. The survey data were linked to employer’s personnel register data for those who gave their written informed consent for linkage and was completed from the surveys for those with no consent for linkage. To ensure data completeness, we included only participants with non-missing values for LTPA and SEP-indicators. This resulted in a final analytical sample of 2,615 participants, of whom 80% were women. A flowchart can be found in supplementary materials (Figure S1).

The data cannot be made publicly available due to strict data protection laws, but access to data can be applied from the Helsinki Health Study group upon reasonable request and following the data sharing policy and data protection laws and regulations.

### Leisure-time physical activity

Participants were asked to report their LTPA over the past year, divided into two main components: activity type and intensity, and time spent per week. Participants were instructed to classify their activities based on the intensity level that best matched their perception of each activity. They then estimated the average weekly duration of LTPA for each intensity level. This structured method allowed us to assess both the intensity level and quantity of LTPA, ensuring a detailed and comprehensive understanding of participants’ LTPA. Physical activities were grouped into levels of intensity (i.e., light, moderate, and vigorous). A metabolic equivalent (MET) measures the energy cost of physical activity, with 1 MET representing the body’s resting energy expenditure. LTPA is typically categorized by intensity: light (< 3 METs), moderate (3–5.9 METs), and vigorous (> 6 METs) [25]. We estimated the total MET hours per week by summing up the volumes of LTPA and further categorizing them by their intensity levels [25–27]. The MET score was handled as a continuous and dichotomous dependent variable in the analysis. If a participant had not provided LTPA in one intensity domain but had provided it in other domains, the LTPA for the missing intensity was assigned a zero value. The cut-off for removing the outliers was determined using the z-score method: a z-score of 3 was considered the threshold [28].

### Childhood socioeconomic indicators

Parental education level and childhood economic difficulties were used as indicators of childhood SEP. In Phase 1, participants reported the highest educational attainment of both of their parents on a four-level scale: lower secondary school or less; vocational school, or equivalent; upper secondary school; and higher education. Parental education (low/high) was classified as high if either the mother or father had attained a higher education degree [29]. Childhood economic difficulties were assessed in Phase 1 by asking the participants whether they had experienced any financial hardships at home before the age of 16 (yes/no) [29].

### Current socioeconomic indicators

Current SEP was assessed based on the participant’s own education level, occupational class, household income, household wealth, and economic difficulties in the Phase 1 survey. Educational attainment was categorized into three levels: high (master’s degree or higher), intermediate (bachelor’s degree), and low (upper secondary school or lower) [29]. Occupational class was classified into three categories: ‘manual or routine non-manual worker’ (e.g., care workers and underground train drivers), ‘semi-professional’ (e.g., nurses and technicians) and ‘professional’ (e.g., teachers, doctors, and managers). Household income was evaluated by estimating the combined disposable income of all the household members, adjusting it for household size according to the modified Organization for Economic Co-operation and Development (OECD) equivalence scale, and then dividing it into tertiles ranging from lowest (1) to highest (3) [30]. Wealth was assessed by asking the participants to estimate the total value of their household property, savings and investments, minus any debts. The responses were classified into 3 groups: ‘<10,000 €’, ‘10,000 €–99,999 €’ and ‘>100,000 €’. Economic difficulties were assessed through two questions: ‘How often do you have enough money to afford the kind of food or clothing you/your family should have? There were five response options indicating difficulties: ‘always’, ‘often’, ‘sometimes’, ‘seldom’, and ‘never’. For questions ‘How much difficulty do you have in meeting the payment of bills?’ response options were: ‘very little or none’, ‘slight’, ‘some’, ‘great’, and ‘very great’ [31]. A sum score was calculated from these responses and divided into three categories: no difficulties (0), occasional difficulties [1–3], and frequent difficulties [4–8, 32].

### Covariates

As covariates, we included key factors that are associated with LTPA and SEP; that is, gender (women/men), age (years), marital status, work status, body mass index (BMI), smoking, and binge drinking, all derived from the Phase 1 survey. The age variable was calculated by subtracting the mean age of participants (32.4 years) from each individual’s age, which was derived from their birth year. This transformed age variable represents the deviation of each participant’s age from the mean age in the study population. Marital status was divided into married/cohabiting vs. other. Work status was categorized to differentiate participants who were temporarily outside the labor market (e.g., due to studying, parental leave, or long-term sickness absence) (“not working”) from those actively “working”. Smoking was divided into smokers (daily and occasional) and non-smokers (non-smokers and ex-smokers). Binge drinking was defined as consuming six or more units of alcohol on a single occasion at least once a week for men and at least once a month for women, as weekly binge drinking was less common among women.

### Statistical analyses

To present the background characteristics of the participants, data were presented as means, medians or as counts with percentages. For descriptive characteristics, the Chi-squared test was performed to assess the statistical significance for categorial variables. For continuous variables, a Mann-Whitney U test was conducted. Furthermore, we used a longitudinal research design with continuous LTPA outcome variable to examine how SEP variables are associated with changes in LTPA over time.

We used generalized linear mixed models (GLMMs) to analyze changes in LTPA intensities over the 5-year follow-up, accounting for repeated observations per participant. Two types of GLMMs were applied: a binomial model (for LTPA participation) and gamma model (for the amount of LTPA, MET-hours/week). In the unadjusted model, MET hours across both phases were used as the dependent variable, separately for different MET levels (light, moderate, vigorous), and the SEP indicators (parental education, current own education, occupational class, childhood and current economic difficulties, household income, and wealth) and phase were used as independent variables as fixed effects. A subject-specific intercept was included as a random effect. This model gives estimates for the baseline MET and the change in MET. In the base model, age and gender were added into the unadjusted model as fixed effects. When analyzing the effects of SEP predictors, each predictor and the predictor’s interaction with phase were added into the base model as fixed effects, thereby adjusting for the effects of the predictor on the baseline MET levels and the change in MET levels separately. To assess how much the predictor explained of the change in MET levels, the effect of phase from the base model was compared to the effect of phase in the model adjusted for the predictor. Further models included also marital status, work status, smoking, binge drinking, and BMI as fixed effects.

To examine whether participants transitioned from engaging in any intensity of LTPA to no activity, we employed binomial GLMMs with a logit link function. This approach allowed us to assess factors associated with complete disengagement from LTPA across different intensity levels. In these models, the dependent variable was a binary indicator (0 MET equals to no engagement, > 0 MET equals to engagement for light, moderate, and vigorous intensity levels) and SEP indicators were the independent variables.

To assess the amount of LTPA (in MET-hours/week), we applied gamma-distributed GLMMs with a log-link function, suitable for positively skewed data. Each LTPA intensity (light, moderate, and vigorous) was analyzed separately, with MET-hours/week as the dependent variable and SEP indicators as the primary independent variables. Since the gamma model with a log-link function does not accommodate zero values, LTPA values of zero were reassigned a value of one to allow for model estimation.

In the binomial GLMM, parameter estimates were exponentiated to exp(β) with 95% confidence intervals (CIs) to provide odds ratios for LTPA participation. In the gamma-distributed GLMM, exp(β) represented mean ratios, indicating the multiplicative effect of the predictors on LTPA levels (MET-hours/week). Furthermore, marginal effects with 95% CIs were computed to estimate the probability of engagement in LTPA (binary outcome) and MET levels (continuous outcome) across SEP groups. In the binary outcome model, probability ratios were derived from marginal effects, while absolute changes over time were assessed using average marginal effects (AMEs). In the continuous outcome model, MET levels were estimated directly, with AMEs representing absolute changes in MET values. Pairwise comparisons were used to test the statistical significance of these changes, and a Šidák correction was applied to adjust for multiple comparisons, ensuring that statistical significance was not inflated due to repeated testing [33].

Interaction analyses revealed no significant differences in the associations between SEP indicators and changes in PA by gender. Furthermore, due to low numbers of men and related uncertainty in the statistical models, our primary analysis was continued with pooled data for women and men. Stata 18.0 (StataCorp LP; College Station, Texas, USA) statistical package was used for all analyses.

## Results

### Participant characteristics at baseline

In the total study population in Phase 1, the mean age was 32.4 years, with men being slightly older and having a higher BMI than women (Table 1). Around two-thirds of participants were married or cohabiting, with men more likely to be married or cohabiting than women. Nearly 90% of participants were working at Phase 1, with a somewhat higher proportion of men working compared to women. More than one in five reported experiencing economic difficulties during childhood. Over half of the participants had faced some level of current economic difficulties, and about 9% had experienced them frequently.

**Table 1:** Descriptive statistics, the Helsinki health Study, 2017 (Phase 1, *n* = 2,615). P-values present differences between women and men

*n*		Overall	Women	Men	*p*
		2615	2102	513	
Age (mean, SD)		32.4 (4.5)	32.2 (4.56)	32.9 (4.38)	0.003
Marital status (n, %)	Married or cohabiting	1729 (66.1)	1362 (64.8)	378 (71.3)	0.004
	Other	886 (33.9)	740 (35.2)	146 (28.5)	
Work status (n, %)	Not working	276 (10.6)	256 (12.2)	20 (3.9)	< 0.001
	Working	2339 (89.4)	1846 (87.8)	493 (96.1)	
Smoking (n, %)	Yes	571 (21.8)	451 (21.5)	120 (23.4)	0.34
	No	2044 (78.2)	1651 (78.5)	393 (76.6)	
Binge drinking (n, %)	Yes	470 (18.0)	392 (18.6)	78 (15.2)	0.07
	No	2145 (82.0)	1710 (81.4)	435 (84.8)	
Body mass index (mean, SD)		25.4 (5.3)	25.2 (5.5)	26.2 (4.4)	< 0.001
Parental education (n, %)	Low	1444 (55.2)	1162 (55.3)	282 (55.0)	0.90
	High	1171 (44.8)	940 (44.7)	231 (45.0)	
Childhood economic difficulties (n, %)	Yes	568 (21.7)	448 (21.3)	120 (23.4)	0.31
	No	2047 (78.3)	1731 (78.7)	393 (76.6)	
Own education (n, %)	Low	755 (28.9)	561 (26.7)	194 (37.8)	< 0.001
	Intermediate	1018 (38.9)	864 (41.1)	154 (30.0)	
	High	842 (32.2)	677 (32.2)	165 (32.2)	
Occupational class (n, %)	Manual/routine non-manual	752 (28.8)	565 (26.7)	187 (36.4)	< 0.001
	Semi-professional	1090 (41.7)	938 (44.6)	152 (29.6)	
	Professional	773 (29.6)	599 (28.5)	174 (33.9)	
Household income (n, %)	1 (lowest)	887 (33.9)	735 (35.0)	152 (29.6)	0.026
	2	954 (36.5)	743 (35.3)	211 (41.1)	
	3 (highest)	774 (29.6)	624 (29.7)	150 (29.3)	
Wealth (n, %)	< 10,000 €	865 (33.1)	737 (35.1)	128 (25.0)	< 0.001
	10,000 €–99,999 €	1082 (41.4)	832 (39.6)	250 (48.7)	
	> 100,000 €	668 (25.5)	533 (25.3)	135 (26.3)	
Current economic difficulties (n, %)	Frequent	234 (8.9)	200 (9.5)	34 (6.6)	0.12
	Occasional	1158 (44.3)	926 (44.1)	232 (45.2)	
	None	1223 (46.8)	976 (46.4)	247 (48.2)	
Leisure-time physical activity engagement (n, %)	Light	2256 (86.3)	1831 (87.1)	425 (82.9)	0.012
	Moderate	1958 (74.9)	1615 (76.8)	343 (66.9)	< 0.001
	Vigorous	1583 (60.5)	1240 (59.0)	343 (66.9)	0.001
Leisure-time physical activity MET (median, IQR)	Light	10.0 (16.0)	11.0 (16.0)	9.0 (14.0)	0.004
	Moderate	7.0 (15.0)	7.8 (15.0)	7.0 (12.0)	< 0.001
	Vigorous	11.0 (28.0)	11.0 (26.0)	17.5 (43.0)	< 0.001

Abbreviations: SD, standard deviation; IQR, interquartile range

Only 28% of the participants had a lower level of education, but men were more likely than women to have lower educational attainment and lower occupational class. Additionally, women were more frequently in the lowest income tertile, and men also had more often wealth above 10,000 €. Women were more likely than men to engage in light- (87% vs. 83%) or moderate-intensity (77% vs. 67%) LTPA and to have higher overall MET scores. In contrast, men participated more in vigorous LTPA (67% vs. 59%) and recorded higher MET levels for this intensity.

Logistic regression models (Supplementary Table S2) showed that attrition was slightly higher among participants with lower parental education (OR = 1.16, 95% CI: 1.03 to 1.31), lower current education (OR = 1.48, 95% CI: 1.26 to 1.73), manual/routine non-manual occupations (OR = 1.31, 95% CI: 1.12 to 1.54), and economic difficulties (OR = 1.34, 95% CI: 1.09 to 1.65). These findings suggest that attrition was somewhat selective with respect to SEP, which may have led to a slight underestimation of socioeconomic differences in LTPA.

**Fig. 1:**
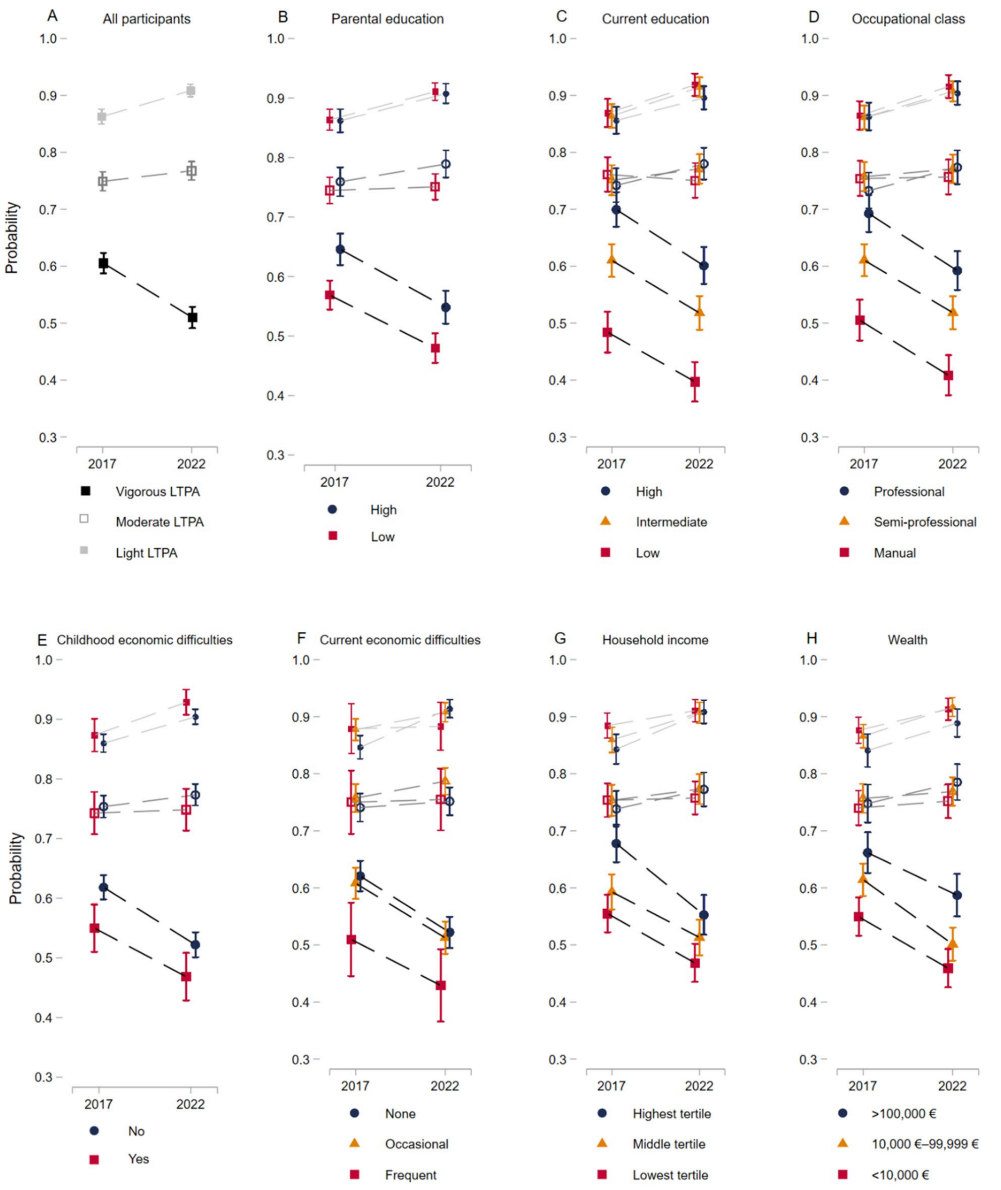
Marginal effects showing the probability rates of engaging in light-, moderate-, or vigorous-intensity leisure-time physical activity (LTPA) in Phases 1 and 2 among the Helsinki Health Study participants (*n* = 2,615). The results are presented for (**A**) all participants, by (**B**) parental education, (**C**) own education, (**D**) occupational class, (**E**) childhood economic difficulties, (**F**) current economic difficulties, (**G**) household income, and (**H**) wealth. The darkest shade represents vigorous-intensity LTPA, the medium shade moderate-intensity LTPA, and the lightest shade light-intensity LTPA. The circle symbol indicates the highest socioeconomic position group, the triangle represents the middle group, and the square represents the lowest group. Average probabilities with their 95% confidence intervals are displayed. Adjusted for gender, age, marital status, work status, body mass index, smoking and binge drinking behavior. The scale has been adjusted to enhance the clarity of the visualization

### Socioeconomic differences in LTPA

#### Engagement in LTPA over the follow-up

Figure 1 presents the probability rates of engaging in light- moderate-, or vigorous intensity LTPA in Phases 1 and 2. The upper panels present the overall sample as well as SEP indicators related to education and occupation, while the lower panels focus on SEP indicators associated with economic factors.

During the follow-up, the probability of engaging in light LTPA increased by 4.6% points (AME = 0.046 [95% CI: 0.03 to 0.06]) in the overall sample. In contrast, engagement in vigorous LTPA decreased by 9.6% points (AME = -0.096 [95% CI: -0.12 to -0.07]). Parental and current education levels, and participants’ occupational class, showed similar declines in vigorous LTPA engagement across groups, with differences persisting between Phase 1 to Phase 2.

Across all SEP indicators, engagement rates in vigorous LTPA decreased, with the largest decline observed among high-income participants (AME = -0.125 [95% CI: -0.14 to -0.03]) compared to middle-income (AME = -0.080 [95% CI: -0.13 to -0.03]) and low-income (AME = -0.087 [95% CI: -0.14 to -0.03]) participants. While differences between high- and low-income groups appeared to narrow slightly during the follow-up, this change was not statistically significant, indicating that disparities remained over time. In contrast, for wealth, the decline in vigorous LTPA participation was most notable in the middle group (High: AME = -0.075 [95% CI: -0.14 to -0.01]; Middle: AME = -0.113 [95% CI: -0.16 to -0.06]; Low: AME = -0.090 [95% CI: -0.15 to -0.03]). Furthermore, differences in vigorous LTPA participation between high- and low-wealth groups were repeated over time.

Overall, engagement rates in vigorous LTPA declined across all SEP groups. However, the change in these differences over time was not statistically significant, suggesting that the disparities between high and low SEP groups persisted throughout the follow-up period, except for childhood economic difficulties, where the statistically significant difference disappeared in Phase 2.

**Fig. 2:**
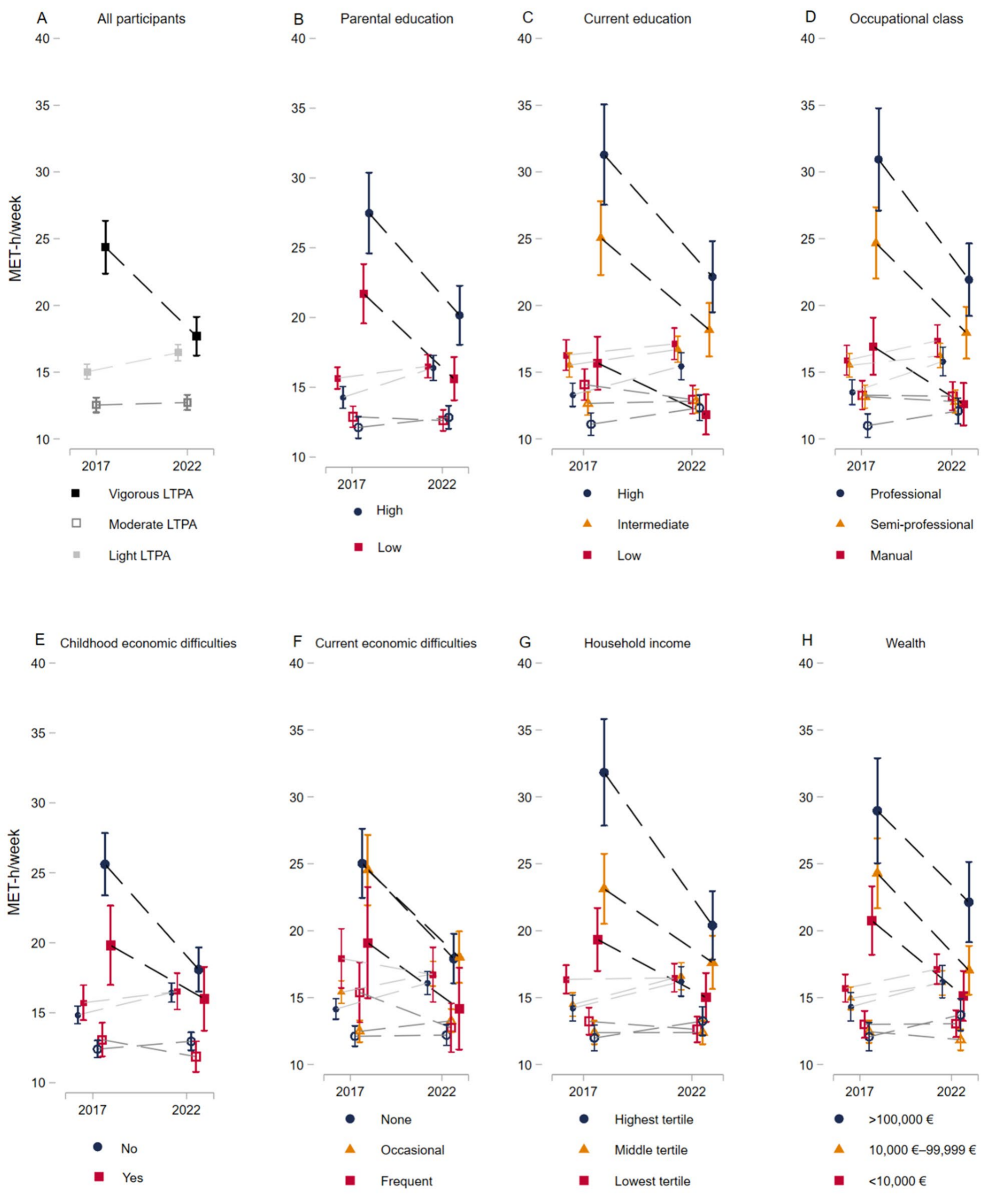
Marginal effects showing values of metabolic equivalent task hours (MET-h) per week for light-, moderate-, or vigorous-intensity leisure-time physical activity (LTPA) in Phases 1 and 2 among the Helsinki Health Study participants (*n* = 2,615). The results are presented for (**A**) all participants, by (**B**) parental education, (**C**) own education, (**D**) occupational class, (**E**) childhood economic difficulties, (**F**) current economic difficulties, (**G**) household income, and (**H**) wealth. The darkest shade represents vigorous-intensity LTPA, the medium shade moderate-intensity LTPA, and the lightest shade light-intensity LTPA. The circle symbol indicates the highest socioeconomic position (SEP) group, the triangle represents the middle group, and the square represents the lowest group. Average MET values with their 95% confidence intervals are displayed. Adjusted for gender, age, marital status, work status, body mass index, smoking and binge drinking behavior. The scale has been adjusted to enhance the clarity of the visualization

#### Changes in LTPA volume over the follow-up

Figure 2 presents the marginal effects of MET-h per week for light-, moderate-, or vigorous intensity LTPA in Phases 1 and 2. During the follow-up, the amount of light LTPA increased by 1.4 MET-h/week (95% CI: 0.7 to 2.2) in the overall sample. Vigorous-intensity LTPA decreased by 6.7 MET-h/week (95% CI: -8.0 to -5.3).

### Education and occupational class

In Phase 1, participants with higher parental education accumulated more vigorous LTPA than those with lower parental education (27.5 MET [95% CI: 24.6 to 30.4] vs. 21.7 MET [95% CI: 19.6 to 23.8]). Similar differences were observed for participants’ own education (high: 31.3 MET [95% CI: 27.5 to 35.1] vs. low: 15.7 MET [95% CI: 13.7 to 17.7]) and occupational class (high: 30.9 MET [95% CI: 27.1 to 34.8] vs. low: 16.9 MET [95% CI: 14.8 to 19.1]). Vigorous LTPA declined across all parental and current education groups as well as occupational class groups. The largest reductions were observed in those with a higher education (-9.2 MET [95% CI: -13.6 to -4.7]) and among those in professional occupations (-9.0 MET [95% CI: -13.6 to -4.4]), whereas the decreases were smaller in lower SEP groups. Consequently, differences in vigorous LTPA between high and low SEP groups narrowed over time for own education (-5.3 MET [95% CI: -8.7 to -1.9]) and occupational class (-4.7 MET [95% CI: -8.2 to -1.2]).

For moderate LTPA, individuals with lower education had higher activity levels at Phase 1 (low: 14.1 MET [95% CI: 12.9 to 15.2] vs. high: 11.1 MET [95% CI: 10.3 to 12.0]), but differences diminished over time as those with higher education increased their MET levels narrowing the differences (-2.4 MET [95% CI: -4.1 to -0.6]). A similar trend was observed in light LTPA and across occupational class groups, though these changes were not statistically significant.

### Economic difficulties

For childhood economic difficulties, individuals without difficulties experienced a greater decline in vigorous LTPA (-7.5 MET [95% CI: -9.7 to -5.4]), leading to a significant reduction in differences between the groups over time (-3.7 MET [95% CI: -6.6 to -0.8]). As a result, the previously statistically significant difference between the groups disappeared in the follow-up. For current economic difficulties, the decline in LTPA was comparable across groups, except for light LTPA, where individuals without economic difficulties increased their activity over the follow-up, narrowing the gap between groups (-3.2 MET [95% CI: -6.1 to -0.2]).

### Household income and wealth

For household income, baseline vigorous LTPA was higher in the high-income group (31.8 MET [95% CI: 27.8 to 35.8]) compared to the low-income group (19.3 MET [95% CI: 17.0 to 21.7]). Furthermore, the decline in vigorous LTPA was greatest in the high-income group (-11.4 MET [95% CI: -16.2 to -6.7]), leading to a substantial reduction in differences between high- and low-income groups over the follow-up (-7.1 MET [95% CI: -10.8 to -3.5]). While this narrowing was statistically significant, disparities between income groups persisted throughout the follow-up, though they gradually diminished over time. In contrast, the high-income group partially compensated for the reduction in vigorous LTPA by increasing their participation in lower-intensity activities, particularly in light LTPA, where differences between income groups narrowed significantly (-1.9 MET [95% CI: -3.6 to -0.2]). For wealth, declines in vigorous LTPA were more comparable across groups, and the differences in its amount between high- and low-wealth groups narrowed only slightly during the follow-up with the change not reaching statistical significance.

Overall, the decline in vigorous LTPA was greater among those without childhood economic difficulties, as well as in higher education, higher occupational class, and high-income groups. This led to a narrowing of differences over the follow-up, though disparities between high and low SEP groups persisted, except for childhood economic difficulties, where the differences disappeared. Some compensation for the decline in vigorous LTPA was observed, with moderate LTPA increasing among the higher-educated and light LTPA increasing among those without economic difficulties and in higher-income groups. All estimates are available in the supplementary material (Supplementary Tables S3–S8).

## Discussion

Our study examined the associations between various life-course SEP indicators and changes in light, moderate, and vigorous LTPA among young and early midlife Finnish employees over a five-year follow-up (2017–2022). The results showed that engagement in vigorous LTPA declined across all SEP groups. However, despite this overall decline, socioeconomic differences largely remained, indicating that reductions in LTPA did not significantly narrow nor widen disparities in LTPA engagement.

When considering MET-based activity levels, the greatest decline in vigorous LTPA was observed among participants with higher education, higher occupational class, and high income. Notably, some compensation for the decline in vigorous LTPA was observed: moderate LTPA increased among the higher-educated, and light LTPA increased among participants without economic difficulties and in higher income groups.

Recent reports on physical activity trends in the Finnish adult population indicate a slight decline in physical activity levels during 2017–19 and 2021–22, as measured in steps per day, with the smallest decrease among 30–39-year-olds [36]. Similarly, our study observed a 6% decline in LTPA (in METs) over the five years, aligning with broader trends despite differences in measurement methods. Furthermore, our findings align with previous research indicating lower overall LTPA participation and intensity levels among lower SEP groups [8, 9, 11, 13, 34–36]. However, there is limited earlier evidence on how changes in different LTPA intensity levels vary across SEP groups [13, 22, 23].

Our results showed that the most notable changes emerged in vigorous LTPA, where the decline was observed across all SEP groups, but was more substantial among the participants with higher SEP. Overall SEP disparities in total LTPA remained somewhat stable, as the participants with higher SEP offset their reduced vigorous LTPA by increasing moderate- and light-intensity activity. A previous large prospective study found that a greater proportion of vigorous LTPA lowered mortality risk the most, even at the same total LTPA levels, while meeting or slightly exceeding moderate LTPA recommendations also reduced mortality [37]. These findings suggest that while overall LTPA levels may remain constant, the relative distribution of different intensity levels can influence health outcomes. In our study, the observed decline in vigorous LTPA among the overall sample highlights the need for long-term follow-up to determine whether this trend is temporary or indicative of a lasting shift in LTPA behavior. This is especially concerning for lower SEP groups, who were already less active at baseline and may face an increased risk of adverse health outcomes [1, 9]. Furthermore, as previous research suggests, lower SEP individuals may also gain the most from increasing their LTPA levels [1].

The finding that income-based differences in LTPA narrowed while wealth-based differences remained more stable suggests that a long-term financial security (i.e. wealth) may play a greater role in maintaining high-intensity exercise than short-term income. Unlike in the income groups, the decline in vigorous LTPA was less pronounced among those with high wealth, leading to more persistent disparities over time. Given that the cohort consists of Finnish public sector employees with relatively small income disparities, the greater decline in vigorous LTPA among the low-wealth group emphasizes the importance of financial stability in sustaining long-term engagement in vigorous activity [17]. In addition, LTPA levels appear to become increasingly associated with income as individuals age, with significant differences emerging by midlife [38].

Furthermore, the similarity in disparity patterns in vigorous LTPA across different SEP indicators, such as education and income, suggests that neither factor alone drives the disparities; rather, each of them may play a crucial role. This finding highlights the multifaceted nature of socioeconomic disparities in LTPA. While education may influence health literacy, awareness of LTPA benefits, and access to structured activities, income may reflect material resources, such as gym memberships, sports equipment, or living environments that facilitate active lifestyles. Moreover, these factors likely interact; for instance, higher education often leads to better-paying jobs, which in turn can provide more opportunities for LTPA [8], while individuals with lower education may face greater financial and other barriers (e.g. lack of time) to participation [39].

Our study took place during a period that coincided with the Covid-19 pandemic, which may partly explain the observed decline in LTPA levels, particularly in vigorous LTPA. Widespread restrictions, such as gym closures, social distancing measures, and limited access to organized sports and recreational facilities, were introduced during the pandemic, all of which likely contributed to the reduction in higher-intensity LTPA. Even if most of the restrictions had been lifted in Finland by 2022 when Phase 2 questionnaire was collected, it is likely that some participants had not returned to their pre-pandemic vigorous activities (e.g. to gyms or organized sports) anymore. A recent review found that during the Covid-19, most studies showed a significant decrease in LTPA, and several studies have shown a reduction particularly in vigorous LTPA [40]. In our study, the decline in vigorous LTPA was greatest among the individuals in the highest SEP groups. This pattern may be explained by the fact that those with higher education, income, or occupational class are more likely to engage in structured, facility-based and often costly, vigorous activities such as gym workouts, group fitness classes, or organized sports [17]—many of which were disrupted by pandemic-related restrictions. In contrast, individuals in lower SEP groups, who may already have had lower engagement in vigorous activity, experienced a relatively smaller decline simply because their baseline levels were lower.

While our study showed that vigorous LTPA generally declined in the entire cohort, it is important to acknowledge that our study population was highly active. However, since the downward trend was consistent across different SEP groups, this suggests that the decline in vigorous LTPA is a widespread phenomenon rather than being confined to specific socioeconomic strata. Adjusting for participants’ own education diminished the effect of parental education on LTPA differences, whereas adding parental education did not alter the effect of participants’ own education. This suggests current SEP plays a stronger role than childhood SEP in shaping LTPA differences. Over the five-year follow-up period, the increase in age may have contributed to the decline in vigorous LTPA, engagement in high-intensity physical activity tends to decrease with age [41, 42]. Furthermore, previous research has found that having children is associated with lower overall physical activity and increases the likelihood of following a declining activity trajectory [38, 43]. Among the participants who were childless in 2017 (*n* = 1527), those who became parents during the five-year follow-up (*n* = 424) showed a larger decline in vigorous LTPA (from 33 to 14 MET, 95% CI: 27.2 to 38.0; 12.0 to 16.8) than those who remained childless (from 24 to 19 MET, 95% CI: 21.6 to 25.8; 17.2 to 20.5). Likewise, participants who reported increased physical (*n* = 595) or mental (*n* = 709) workload showed greater decreases (physical: from 24 to 14 MET, 95% CI: 20.2 to 26.9; 12.0 to 16.0; vs. from 25 to 20 MET, 95% CI: 23.0 to 27.6; 17.7 to 21.3; mental: from 24 to 16 MET, 95% CI: 20.4 to 26.6; 13.7 to 18.0; vs. from 26 to 19 MET, 95% CI: 23.2 to 28.0; 17.3 to 20.8). Increased physical or mental workload can contribute to reduced engagement in vigorous LTPA, primarily due to heightened fatigue and diminished recovery capacity [44, 45].

However, the fact that the trend was consistent across both models reinforces the robustness of our findings and suggests that the decline is not only a reduction in frequency or duration but, for a significant portion of individuals, a complete disengagement from vigorous exercise. Furthermore, it is particularly concerning that participants in lower SEP groups engaged significantly less in vigorous-intensity LTPA, which is known to provide substantial health benefits [3, 46].

### Methodological considerations

Several limitations of this study should be acknowledged. The study participants were all public sector employees, 80% of whom were women, reflecting the general composition of Finnish public sector workers and the target population [47]. This limits the generalizability of the findings. Although disparities between high and low SEP groups narrowed, longer follow-up periods are necessary to determine whether the decline in vigorous LTPA is a persistent trend or part of natural fluctuations in LTPA over the life-course. Changes in LTPA behavior during life transitions, such as retirement, tend to be more favorable for high SEP adults, suggesting that socioeconomic factors influence physical activity patterns over time [23, 48]. Additionally, the use of self-reported data may introduce information bias, which may lead to overestimation of activity levels (recall bias). Social desirability bias could also be a factor, as the participants may have provided answers they perceived as favorable, leading to potential overreporting [49, 50]. Most of the participants across all SEP groups reported levels of LTPA that exceeded the recommended thresholds of moderate or vigorous LTPA (7.5–15 MET-h/week). In addition, an important consideration is whether the observed changes in LTPA truly reflect absolute changes in LTPA levels within the cohort or whether they are influenced by selection effects. Furthermore, LTPA was self-reported for the past year, which may be affected by seasonal variation typical to Finland. However, the measure aimed to capture habitual activity patterns rather than short-term fluctuations, and any random error due to seasonal variation is likely to have attenuated rather than inflated the observed associations. In addition, the one-year recall period was used to capture habitual and long-term physical activity rather than short-term fluctuations, and it is a commonly applied approach in large epidemiological studies [51, 52]. Nevertheless, in our study, individuals who did not respond in Phase 2 were slightly less active in Phase 1 compared to those who responded, which supports the interpretation that the decline in vigorous LTPA could reflect a true behavioral shift rather than merely being a consequence of differential attrition. Another potential explanation for the magnitude of this decline could be regression to the mean-effect, wherein individuals who initially reported very high levels show a decrease over time, even without an actual behavioral change [53]. The occupational class variable was based on the City of Helsinki’s personnel register rather than the international ISCO classification. However, the classification follows similar principles regarding job tasks, qualification and skill level, and has been consistently used in all previous HHS publications, ensuring comparability across study waves [54].

Our study has also several strengths. By including both binary and continuous approaches, we were able to illustrate the phenomenon from two complementary perspectives. The continuous measure provided a concrete estimate of the magnitude of change in LTPA, capturing absolute reductions in activity levels, while the binary classification highlighted how many participants transitioned from engaging in certain intensity level of LTPA to no longer participating. The consistency of the trend across both models strengthens the interpretation that the decline in vigorous LTPA is widespread and affects individuals regardless of SEP. Moreover, we examined a large employee cohort including both women and men and representing a wide range of occupations across different SEP levels. We measured LTPA data at two time points and with different intensities, investigating prospective relationships and changes in LTPA over the study period. We utilized a GLMM approach to account for individual-level variability. This strengthens the model’s ability to estimate the effects of SEP on LTPA over time with greater accuracy.

Future research could further examine whether those who decrease or completely discontinue vigorous LTPA are likely to re-engage or increase vigorous LTPA over time, or whether this represents a more permanent behavioral shift, particularly in light of broader societal and Covid-19-pandemic-related changes in LTPA habits. Understanding these dynamics will be crucial for developing targeted interventions that encourage the return to higher-intensity LTPA across all SEP levels.

In conclusion, this study among young and early midlife public sector employees revealed an overall decline in vigorous LTPA levels across all life-course SEP groups, with engagement rates decreasing and differences between higher and lower SEP groups persisting over a 5-year period. Moreover, while higher SEP individuals may have compensated for the decline in vigorous LTPA by increasing their light or moderate activity, those in lower SEP groups did not show the same compensatory adaptation. Furthermore, our findings suggest that current SEP plays a stronger role than childhood SEP in shaping LTPA differences. Our study highlights the persistence of socioeconomic differences in LTPA among Finnish young and early midlife employees. Therefore, in efforts to increase LTPA and narrow the socioeconomic differences in LTPA, targeted interventions that specifically aim to increase LTPA among more disadvantaged employee groups is warranted. Our results raise concerns about the long-term health and work ability effects of overall declining vigorous LTPA, particularly for lower SEP individuals who already engage in less vigorous LTPA.

## Supplementary Information

Below is the link to the electronic supplementary material.

Supplementary Material 1

## Data Availability

The data cannot be made publicly available due to strict data protection laws, but access to data can be applied from the Helsinki Health Study group upon reasonable request and following the data sharing policy and data protection laws and regulations.
