# Supplementary material for: Life-course socioeconomic circumstances and changes in leisure-time physical activity among young and early midlife employees
Source: Int J Equity Health. 2026 Jan 30;25:54. doi: 10.1186/s12939-026-02769-3 (PMC12930950; doi:10.1186/s12939-026-02769-3)
Supplement: Supplementary file 1 — Supplementary Material 1 [file 12939_2026_2769_MOESM1_ESM.docx]

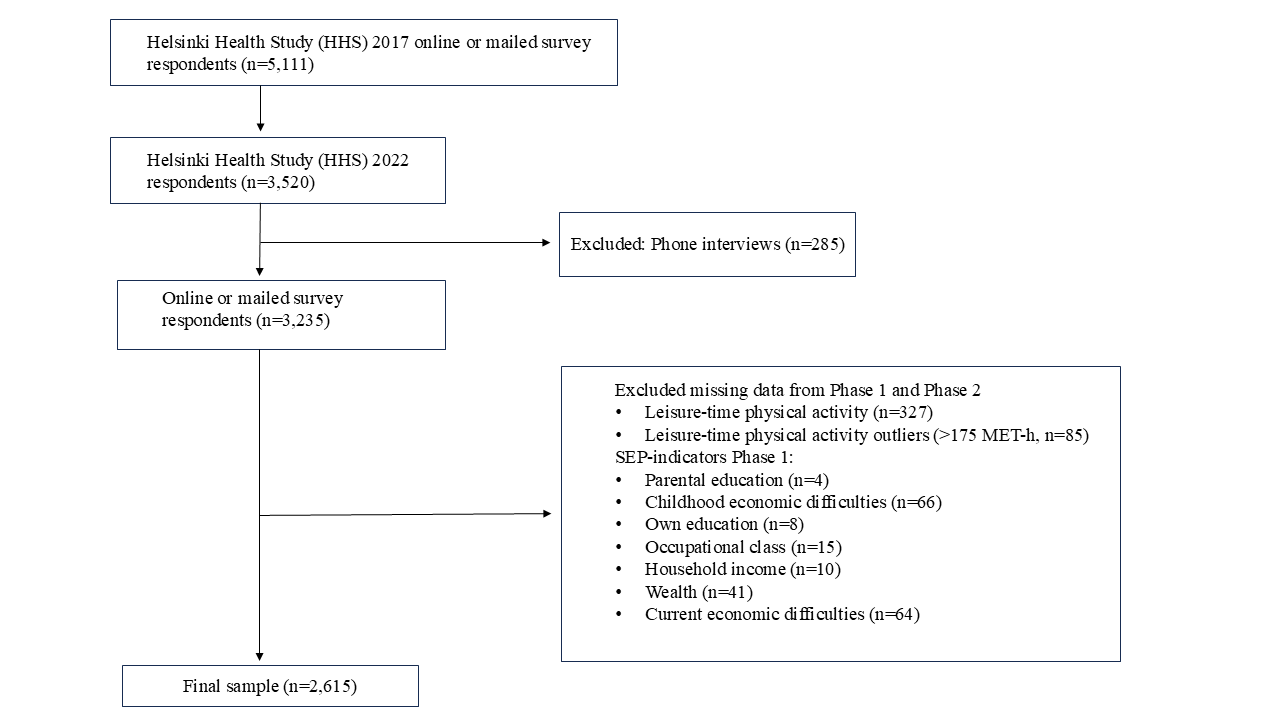


**Supplementary Figure S1** A flowchart of the Helsinki Health Study (HHS) participants 2017–2022 (n=2,615).

**Supplementary Table S2.** Logistic regression for attrition (lost to follow-up, yes=1 / no=0) according to socioeconomic position (SEP) indicators and leisure-time physical activity among the Helsinki Health Study participants (n=4,443). Odds ratios (95% CI), adjusted for gender and age.

| SEP indicator | Category (ref = highest SEP) | OR (95% CI) |
| --- | --- | --- |
| Parental education | Low vs. high | 1.16 (1.03–1.31) |
| Own education | Low vs. high | 1.48 (1.26–1.73) |
|  | Intermediate vs. high | 1.03 (0.88–1.20) |
| Occupational class | Manual vs. professional | 1.31 (1.12–1.54) |
|  | Semi-pro vs. professional | 0.99 (0.85–1.15) |
| Childhood economic difficulties | Yes vs. no | 0.97 (0.84–1.13) |
| Current economic difficulties | Frequent vs. none | 1.34 (1.09–1.65) |
|  | Occasional vs. none | 1.10 (0.97–1.25) |
| Household income | Lowest vs. highest tertile | 0.92 (0.79–1.07) |
|  | Middle vs. highest tertile | 0.85 (0.73–0.98) |
| Wealth | Low vs. high | 1.03 (0.88–1.22) |
|  | Middle vs. high | 0.92 (0.79–1.08) |
| Leisure-time physical activity | Low vs high | 1.02 (0.89–1.17) |

**Supplementary Table S3** Marginal effects of the probability rates of engaging in vigorous-intensity leisure-time physical activity (LTPA) in Phases 1 and 2 among the Helsinki Health Study participants (n=2,615). The estimates are exponentiated to exp(β) with 95% confidence intervals (CI) to provide odds ratios for LTPA participation between phases. Absolute changes over time and between low and high SEP groups are presented with average marginal effects (AMEs) with 95% CIs. Adjusted for gender, age, marital status, work status, body mass index, smoking and binge drinking behavior.

|  | Phase 1 LTPA | | Phase 2 LTPA | | Change Phase 1–2 |  | Low vs. High Change | |  |
| --- | --- | --- | --- | --- | --- | --- | --- | --- | --- |
|  | exp(β) | 95% CI | exp(β) | 95% CI | AME | 95% CI | AME | 95% CI |  |
| Overall sample | 0.61 | 0.59–0.62 | 0.51 | 0.49–0.53 | -0.096 | -0.12– -0.07 |  |  |  |
| Parental education |  |  |  |  |  |  |  |  |  |
| Low | 0.57 | 0.55–0.60 | 0.48 | 0.45–0.50 | -0.091 | -0.13– -0.05 |  |  |  |
| High | 0.65 | 0.62–0.67 | 0.55 | 0.52–0.57 | -0.101 | -0.14– -0.06 | 0.001 | -0.05–0.03 |  |
| Own education |  |  |  |  |  |  |  |  |  |
| Low | 0.49 | 0.45–0.52 | 0.40 | 0.36–0.43 | -0.090 | -0.15– -0.03 |  |  |  |
| Intermediate | 0.61 | 0.58–0.64 | 0.52 | 0.49–0.55 | -0.094 | -0.14– -0.04 |  |  |  |
| High | 0.70 | 0.67–0.73 | 0.60 | 0.57–0.63 | -0.102 | -0.16– -0.05 | -0.012 | -0.07–0.04 |  |
| Occupational class |  |  |  |  |  |  |  |  |  |
| Manual/routine non-manual | 0.51 | 0.47–0.54 | 0.41 | 0.37–0.44 | -0.097 | -0.16– -0.04 |  |  |  |
| Semi-professional | 0.61 | 0.58–0.64 | 0.52 | 0.49–0.55 | -0.092 | -0.14– -0.04 |  |  |  |
| Professional | 0.69 | 0.66–0.72 | 0.59 | 0.56–0.63 | -0.100 | -0.16– -0.04 | -0.004 | -0.06–0.05 |  |
| Household income |  |  |  |  |  |  |  |  |  |
| 1 (lowest) | 0.56 | 0.52–0.59 | 0.47 | 0.44–0.50 | -0.087 | -0.14– -0.03 |  |  |  |
| 2 | 0.59 | 0.56–0.62 | 0.51 | 0.48–0.54 | -0.080 | -0.13– -0.03 |  |  |  |
| 3 (highest) | 0.68 | 0.65–0.71 | 0.55 | 0.52–0.58 | -0.125 | -0.18– -0.07 | -0.038 | -0.09–0.02 |  |
| Wealth |  |  |  |  |  |  |  |  |  |
| <10,000 € | 0.55 | 0.52–0.58 | 0.46 | 0.43–0.49 | -0.090 | -0.15– -0.04 |  |  |  |
| 10,000 €–99,999 € | 0.61 | 0.59–0.64 | 0.50 | 0.47–0.53 | -0.113 | -0.16– -0.06 |  |  |  |
| >100,000 € | 0.66 | 0.63–0.70 | 0.59 | 0.55–0.62 | -0.075 | -0.14– -0.01 | 0.016 | -0.04–0.07 |  |
| Current economic difficulties |  |  |  |  |  |  |  |  |  |
| Frequent | 0.51 | 0.45–0.57 | 0.43 | 0.36–0.49 | -0.081 | -0.19–0.03 |  |  |  |
| Occasional | 0.61 | 0.58–0.64 | 0.51 | 0.49–0.54 | -0.095 | -0.14– -0.05 |  |  |  |
| None | 0.62 | 0.59–0.65 | 0.52 | 0.49–0.55 | -0.099 | -0.15– -0.05 | 0.018 | -0.06–0.10 |  |

**Supplementary Table S4** Marginal effects of the probability rates of engaging in moderate-intensity leisure-time physical activity (LTPA) in Phases 1 and 2 among the Helsinki Health Study participants (n=2,615). The estimates are exponentiated to exp(β) with 95% confidence intervals (CI) to provide odds ratios for LTPA participation between phases. Absolute changes over time and between low and high SEP groups are presented with average marginal effects (AMEs) with 95% CIs. Adjusted for gender, age, marital status, work status, body mass index, smoking and binge drinking behavior.

|  | Phase 1 | | Phase 2 | | Change Phase 1–2 |  | Low vs. High Change | |  |
| --- | --- | --- | --- | --- | --- | --- | --- | --- | --- |
|  | exp(β) | 95% CI | exp(β) | 95% CI | AME | 95% CI | AME | 95% CI |  |
| Overall sample | 0.75 | 0.73–0.77 | 0.77 | 0.75–0.78 | 0.019 | -0.01–0.04 |  |  |  |
| Parental education |  |  |  |  |  |  |  |  |  |
| Low | 0.74 | 0.72–0.77 | 0.75 | 0.73–0.77 | 0.008 | -0.03–0.05 |  |  |  |
| High | 0.76 | 0.73–0.78 | 0.55 | 0.76–0.81 | 0.031 | -0.01–0.07 | 0.023 | -0.02–0.06 |  |
| Own education |  |  |  |  |  |  |  |  |  |
| Low | 0.76 | 0.73–0.79 | 0.75 | 0.72–0.78 | -0.008 | -0.07–0.05 |  |  |  |
| Intermediate | 0.75 | 0.72–0.78 | 0.77 | 0.75–0.80 | 0.021 | -0.03–0.07 |  |  |  |
| High | 0.74 | 0.71–0.77 | 0.78 | 0.75–0.81 | 0.040 | -0.01–0.09 | 0.048 | -0.01–0.10 |  |
| Occupational class |  |  |  |  |  |  |  |  |  |
| Manual/routine non-manual | 0.75 | 0.72–0.79 | 0.76 | 0.73–0.79 | 0.003 | -0.05–0.06 |  |  |  |
| Semi-professional | 0.76 | 0.73–0.78 | 0.77 | 0.75–0.80 | 0.014 | -0.03–0.06 |  |  |  |
| Professional | 0.73 | 0.70–0.76 | 0.77 | 0.74–0.80 | 0.041 | -0.02–0.10 | 0.038 | -0.02–0.09 |  |
| Household income |  |  |  |  |  |  |  |  |  |
| 1 (lowest) | 0.75 | 0.72–0.78 | 0.76 | 0.73–0.79 | 0.004 | -0.05–0.06 |  |  |  |
| 2 | 0.75 | 0.73–0.78 | 0.75 | 0.75–0.80 | 0.019 | -0.03–0.07 |  |  |  |
| 3 (highest) | 0.74 | 0.71–0.77 | 0.77 | 0.74–0.80 | 0.034 | -0.02–0.09 | 0.030 | -0.02–0.08 |  |
| Wealth |  |  |  |  |  |  |  |  |  |
| <10,000 € | 0.74 | 0.71–0.77 | 0.75 | 0.72–0.78 | 0.012 | -0.04–0.07 |  |  |  |
| 10,000 €–99,999 € | 0.76 | 0.73–0.78 | 0.77 | 0.74–0.79 | 0.012 | -0.04–0.06 |  |  |  |
| >100,000 € | 0.75 | 0.71–0.78 | 0.79 | 0.75–0.82 | 0.037 | -0.02–0.10 | 0.025 | -0.03–0.08 |  |
| Current economic difficulties |  |  |  |  |  |  |  |  |  |
| Frequent | 0.75 | 0.69–0.81 | 0.75 | 0.70–0.81 | 0.005 | -0.01–0.11 |  |  |  |
| Occasional | 0.76 | 0.73–0.78 | 0.79 | 0.76–0.81 | 0.029 | -0.02–0.07 |  |  |  |
| None | 0.74 | 0.72–0.77 | 0.75 | 0.73–0.78 | 0.011 | -0.03–0.06 | -0.006 | -0.08–0.07 |  |

**Supplementary Table S5** Marginal effects of the probability rates of engaging in light-intensity leisure-time physical activity (LTPA) in Phases 1 and 2 among the Helsinki Health Study participants (n=2,615). The estimates are exponentiated to exp(β) with 95% confidence intervals (CI) to provide odds ratios for LTPA participation between phases. Absolute changes over time and between low and high SEP groups are presented with average marginal effects (AMEs) with 95% CIs. Adjusted for gender, age, marital status, work status, body mass index, smoking and binge drinking behavior.

|  | Phase 1 | | Phase 2 | | Change Phase 1–2 |  | Low vs. High Change | |  |
| --- | --- | --- | --- | --- | --- | --- | --- | --- | --- |
|  | exp(β) | 95% CI | exp(β) | 95% CI | AME | 95% CI | AME | 95% CI |  |
| Overall sample | 0.86 | 0.85–0.88 | 0.91 | 0.90–0.92 | 0.046 | 0.03–0.06 |  |  |  |
| Parental education |  |  |  |  |  |  |  |  |  |
| Low | 0.86 | 0.85–0.88 | 0.91 | 0.90–0.93 | 0.047 | 0.02–0.08 |  |  |  |
| High | 0.86 | 0.84–0.88 | 0.91 | 0.89–0.92 | 0.044 | 0.01–0.08 | -0.003 | -0.04–0.03 |  |
| Own education |  |  |  |  |  |  |  |  |  |
| Low | 0.87 | 0.85–0.90 | 0.92 | 0.90–0.94 | 0.046 | 0.01–0.09 |  |  |  |
| Intermediate | 0.86 | 0.84–0.88 | 0.91 | 0.90–0.93 | 0.050 | 0.01–0.09 |  |  |  |
| High | 0.86 | 0.83–0.88 | 0.90 | 0.88–0.92 | 0.041 | -0.01–0.08 | -0.005 | -0.05–0.04 |  |
| Occupational class |  |  |  |  |  |  |  |  |  |
| Manual/routine non-manual | 0.86 | 0.84–0.89 | 0.92 | 0.90–0.94 | 0.051 | 0.01–0.10 |  |  |  |
| Semi-professional | 0.86 | 0.84–0.88 | 0.91 | 0.89–0.92 | 0.046 | 0.01–0.08 |  |  |  |
| Professional | 0.86 | 0.84–0.89 | 0.90 | 0.88–0.92 | 0.041 | -0.01–0.09 | -0.010 | -0.05–0.03 |  |
| Household income |  |  |  |  |  |  |  |  |  |
| 1 (lowest) | 0.88 | 0.86–0.91 | 0.91 | 0.89–0.93 | 0.026 | -0.01–0.07 |  |  |  |
| 2 | 0.86 | 0.84–0.88 | 0.91 | 0.89–0.93 | 0.048 | 0.01–0.09 |  |  |  |
| 3 (highest) | 0.84 | 0.82–0.87 | 0.91 | 0.89–0.93 | 0.066 | 0.02–0.11 | 0.040 | -0.01–0.08 |  |
| Wealth |  |  |  |  |  |  |  |  |  |
| <10,000 € | 0.88 | 0.85–0.90 | 0.91 | 0.89–0.93 | 0.037 | -0.01–0.08 |  |  |  |
| 10,000 €–99,999 € | 0.87 | 0.85–0.89 | 0.92 | 0.90–0.93 | 0.051 | 0.01–0.09 |  |  |  |
| >100,000 € | 0.84 | 0.71–0.78 | 0.89 | 0.86–0.91 | 0.048 | -0.01–0.10 | 0.011 | -0.03–0.06 |  |
| Current economic difficulties |  |  |  |  |  |  |  |  |  |
| Frequent | 0.88 | 0.84–0.93 | 0.88 | 0.84–0.93 | 0.004 | -0.08–0.09 |  |  |  |
| Occasional | 0.88 | 0.86–0.90 | 0.91 | 0.89–0.92 | 0.030 | -0.01–0.07 |  |  |  |
| None | 0.85 | 0.83–0.87 | 0.91 | 0.90–0.93 | 0.068 | 0.03–0.10 | -0.063 | -0.13– -0.01 |  |

**Supplementary Table S6** Average metabolic equivalent task hours (MET-h) per week for vigorous-intensity leisure-time physical activity (LTPA) in Phases 1 and 2 among the Helsinki Health Study participants (n=2,615). The estimates are exponentiated to exp(β) with 95% confidence intervals (CI) to provide mean ratios for LTPA participation between phases. Absolute changes over time and between low and high SEP groups are presented with average marginal effects (AMEs) with 95% CIs. Adjusted for gender, age, marital status, work status, body mass index, smoking and binge drinking behavior.

|  | Phase 1 LTPA | | Phase 2 LTPA | | Change Phase 1–2 |  | Low vs. High Change | |  |
| --- | --- | --- | --- | --- | --- | --- | --- | --- | --- |
|  | exp(β) | 95% CI | exp(β) | 95% CI | AME | 95% CI | AME | 95% CI |  |
| Overall sample | 24.37 | 22.4–26.4 | 17.69 | 16.3–19.1 | -6.68 | -8.0– -5.3 |  |  |  |
| Parental education |  |  |  |  |  |  |  |  |  |
| Low | 21.70 | 19.6–23.8 | 15.61 | 14.1–17.2 | -6.09 | -8.3– -3.9 |  |  |  |
| High | 27.48 | 24.6–30.4 | 20.16 | 18.1–22.3 | -7.31 | -10.4– -4.3 | -1.22 | -4.0–1.5 |  |
| Own education |  |  |  |  |  |  |  |  |  |
| Low | 15.68 | 13.7–17.7 | 11.83 | 10.3–13.3 | -3.86 | -6.3– -1.4 |  |  |  |
| Intermediate | 25.05 | 22.3–27.8 | 18.17 | 16.2–20.2 | -6.87 | -10.2– -3.6 |  |  |  |
| High | 31.29 | 27.5–35.1 | 22.14 | 19.5–24.8 | -9.15 | -13.6– -4.7 | -5.29 | -8.7– -1.9 |  |
| Occupational class |  |  |  |  |  |  |  |  |  |
| Manual/routine non-manual | 16.93 | 14.8–19.1 | 12.60 | 11.0–14.2 | -4.33 | -6.9– -1.7 |  |  |  |
| Semi-professional | 24.67 | 22.0–27.3 | 17.96 | 16.0–19.9 | -6.71 | -9.9– -3.5 |  |  |  |
| Professional | 30.94 | 27.1–34.8 | 21.93 | 19.2–24.6 | -9.01 | -13.6– -4.4 | -4.68 | -8.2– -1.2 |  |
| Household income |  |  |  |  |  |  |  |  |  |
| 1 (lowest) | 19.34 | 17.0–21.7 | 15.02 | 13.2–16.8 | -4.32 | -7.1– -1.5 |  |  |  |
| 2 | 23.13 | 20.5–25.7 | 17.63 | 15.6–19.6 | -5.49 | -8.6– -2.3 |  |  |  |
| 3 (highest) | 31.82 | 27.8–35.8 | 20.39 | 17.8–22.9 | -11.4 | -16.2– -6.7 | -7.10 | -10.8– -3.5 |  |
| Wealth |  |  |  |  |  |  |  |  |  |
| <10,000 € | 20.75 | 18.2–23.3 | 15.11 | 13.3–17.0 | -5.64 | -8.6– -2.6 |  |  |  |
| 10,000 €–99,999 € | 24.28 | 21.7–26.9 | 17.04 | 15.2–18.9 | -7.24 | -10.3– -4.2 |  |  |  |
| >100,000 € | 28.96 | 25.0–32.9 | 22.13 | 19.1–25.1 | -6.83 | -11.5– -2.1 | -1.19 | -4.9–2.5 |  |
| Current economic difficulties |  |  |  |  |  |  |  |  |  |
| Frequent | 19.09 | 14.9–23.2 | 14.17 | 11.1–17.2 | -4.92 | -10.3–0.5 |  |  |  |
| Occasional | 24.53 | 21.9–27.1 | 18.01 | 16.1–19.9 | -6.51 | -9.5– -3.5 |  |  |  |
| None | 25.02 | 22.4–27.6 | 17.91 | 16.1–19.8 | -7.12 | -10.1– -4.1 | 2.20 | -1.9–6.3 |  |

**Supplementary Table S7** Average metabolic equivalent task hours (MET-h) per week for moderate-intensity leisure-time physical activity (LTPA) in Phases 1 and 2 among the Helsinki Health Study participants (n=2,615). The estimates are exponentiated to exp(β) with 95% confidence intervals (CI) to provide mean ratios for LTPA participation between phases. Absolute changes over time and between low and high SEP groups are presented with average marginal effects (AMEs) with 95% CIs. Adjusted for gender, age, marital status, work status, body mass index, smoking and binge drinking behavior.

|  | Phase 1 LTPA | | Phase 2 LTPA | | Change Phase 1–2 |  | Low vs. High Change | |  |
| --- | --- | --- | --- | --- | --- | --- | --- | --- | --- |
|  | exp(β) | 95% CI | exp(β) | 95% CI | AME | 95% CI | AME | 95% CI |  |
| Overall sample | 12.55 | 12.0–13.1 | 12.73 | 12.2–13.3 | 0.18 | -0.5–0.9 |  |  |  |
| Parental education |  |  |  |  |  |  |  |  |  |
| Low | 12.90 | 12.1–13.7 | 12.62 | 11.9–13.4 | -0.27 | -1.5–1.0 |  |  |  |
| High | 12.12 | 11.3–12.9 | 12.84 | 12.0–13.7 | 0.72 | -0.6–2.0 | 0.99 | -0.4–2.3 |  |
| Own education |  |  |  |  |  |  |  |  |  |
| Low | 14.07 | 12.9–15.2 | 12.94 | 11.9–14.0 | -1.13 | -3.1–0.9 |  |  |  |
| Intermediate | 12.66 | 11.8–27.8 | 12.84 | 12.0–13.7 | 0.17 | -1.4–1.8 |  |  |  |
| High | 11.10 | 10.3–12.0 | 12.33 | 11.4–13.3 | 1.23 | -0.4–2.9 | 2.36 | 0.6–4.1 |  |
| Occupational class |  |  |  |  |  |  |  |  |  |
| Manual/routine non-manual | 13.28 | 12.2–19.1 | 13.20 | 12.1–14.3 | -0.07 | -2.1–1.9 |  |  |  |
| Semi-professional | 13.17 | 12.3–14.0 | 12.80 | 11.9–13.6 | -0.37 | -2.0–1.2 |  |  |  |
| Professional | 11.00 | 10.1–11.9 | 12.10 | 11.1–13.1 | 1.10 | -0.6–2.8 | 1.17 | -0.6–2.9 |  |
| Household income |  |  |  |  |  |  |  |  |  |
| 1 (lowest) | 13.23 | 12.2–14.2 | 12.62 | 11.7–13.6 | -0.61 | -2.4–1.6 |  |  |  |
| 2 | 12.38 | 11.5–13.3 | 12.39 | 11.5–13.3 | 0.01 | -1.6–1.6 |  |  |  |
| 3 (highest) | 11.99 | 11.0–12.9 | 13.25 | 12.2–14.3 | 1.26 | -0.6–3.1 | 1.87 | 0.2–3.6 |  |
| Wealth |  |  |  |  |  |  |  |  |  |
| <10,000 € | 13.00 | 12.0–14.0 | 13.05 | 12.1–14.0 | 0.05 | -1.8–1.9 |  |  |  |
| 10,000 €–99,999 € | 12.46 | 11.6–13.3 | 11.86 | 11.1–12.7 | -0.60 | -2.1–0.9 |  |  |  |
| >100,000 € | 12.07 | 25.0–32.9 | 13.71 | 12.5–14.9 | 1.64 | -0.4–3.7 | 1.59 | -0.3–3.4 |  |
| Current economic difficulties |  |  |  |  |  |  |  |  |  |
| Frequent | 15.40 | 13.2–17.6 | 12.75 | 10.9–14.6 | -2.64 | -6.5–1.2 |  |  |  |
| Occasional | 12.48 | 11.7–13.3 | 13.27 | 12.4–14.1 | 0.78 | -0.7–2.3 |  |  |  |
| None | 12.11 | 11.3–12.9 | 12.20 | 11.4–13.0 | 0.09 | -1.3–1.5 | -2.73 | -5.5–0.1 |  |

**Supplementary Table S8** Average metabolic equivalent task hours (MET-h) per week for light-intensity leisure-time physical activity (LTPA) in Phases 1 and 2 among the Helsinki Health Study participants (n=2,615). The estimates are exponentiated to exp(β) with 95% confidence intervals (CI) to provide mean ratios for LTPA participation between phases. Absolute changes over time and between low and high SEP groups are presented with average marginal effects (AMEs) with 95% CIs. Adjusted for gender, age, marital status, work status, body mass index, smoking and binge drinking behavior.

|  | Phase 1 LTPA | | Phase 2 LTPA | | Change Phase 1–2 |  | Low vs. High Change | |  |
| --- | --- | --- | --- | --- | --- | --- | --- | --- | --- |
|  | exp(β) | 95% CI | exp(β) | 95% CI | AME | 95% CI | AME | 95% CI |  |
| Overall sample | 15.03 | 14.5–15.6 | 16.46 | 15.8–17.1 | 1.43 | 0.7–2.2 |  |  |  |
| Parental education |  |  |  |  |  |  |  |  |  |
| Low | 15.66 | 14.9–16.4 | 16.51 | 15.7–17.3 | 0.85 | -0.5–2.2 |  |  |  |
| High | 14.28 | 13.5–15.1 | 16.40 | 15.5–17.3 | 2.12 | 0.7–3.6 | 1.26 | -0.2–2.7 |  |
| Own education |  |  |  |  |  |  |  |  |  |
| Low | 16.28 | 15.2–17.4 | 17.12 | 15.9–18.3 | 0.84 | -1.3–3.0 |  |  |  |
| Intermediate | 15.53 | 14.6–16.4 | 16.71 | 15.7–17.7 | 1.19 | -0.6–3.0 |  |  |  |
| High | 13.30 | 12.4–14.2 | 15.45 | 14.4–16.5 | 2.15 | 0.4–3.9 | 1.30 | -0.6–3.2 |  |
| Occupational class |  |  |  |  |  |  |  |  |  |
| Manual/routine non-manual | 15.90 | 14.8–17.0 | 17.36 | 16.2–18.5 | 1.45 | -0.7–3.6 |  |  |  |
| Semi-professional | 15.51 | 14.6–16.4 | 16.24 | 15.3–17.2 | 0.73 | -1.0–2.4 |  |  |  |
| Professional | 13.50 | 12.6–14.4 | 15.81 | 14.7–16.9 | 2.31 | -0.4–4.2 | 0.85 | -1.1–2.8 |  |
| Household income |  |  |  |  |  |  |  |  |  |
| 1 (lowest) | 16.36 | 12.2–14.2 | 16.48 | 11.7–13.6 | 0.12 | -1.9–2.1 |  |  |  |
| 2 | 14.48 | 11.5–13.3 | 16.60 | 11.5–13.3 | 2.12 | 0.3–3.9 |  |  |  |
| 3 (highest) | 14.22 | 11.0–12.9 | 16.21 | 12.2–14.3 | 1.99 | 0.1–3.9 | 1.87 | 0.1–3.7 |  |
| Wealth |  |  |  |  |  |  |  |  |  |
| <10,000 € | 15.69 | 14.7–16.7 | 17.12 | 16.0–18.2 | 1.42 | -0.6–3.4 |  |  |  |
| 10,000 €–99,999 € | 14.94 | 14.1–15.8 | 16.09 | 15.2–17.0 | 1.15 | -0.5–2.8 |  |  |  |
| >100,000 € | 14.31 | 13.2–15.4 | 16.18 | 15.0–17.4 | 1.88 | -0.2–4.0 | 0.45 | -1.5–2.4 |  |
| Current economic difficulties |  |  |  |  |  |  |  |  |  |
| Frequent | 17.93 | 15.7–20.2 | 16.71 | 14.7–18.8 | -1.21 | -5.3–2.8 |  |  |  |
| Occasional | 15.40 | 14.6–16.3 | 16.79 | 15.9–17.7 | 1.38 | -0.3–3.1 |  |  |  |
| None | 14.15 | 13.4–14.9 | 16.09 | 15.2–17.0 | 1.93 | 0.4–3.5 | -3.15 | -6.1– -0.2 |  |
